# Prevalence of burnout and its determinants among Indonesian nurses: a multicentre study

**DOI:** 10.1038/s41598-024-63550-6

**Published:** 2024-12-30

**Authors:** I. Gede Juanamasta, Yupin Aungsuroch, Joko Gunawan, Michael Joseph Dino, Rapin Polsook

**Affiliations:** 1 Faculty of Nursing, Chulalongkorn University, Borommaratchachonnani Srisataphat, Building, Rama 1 Road, Pathumwan, 10330 Bangkok Thailand; 2 Alice Lee Centre for Nursing Studies, National University of Singapore, Singapore, Singapore; 3 Research Development and Innovation Centre, Our Lady Fatima University, Valenzuela, Philippines

**Keywords:** Burnout, Hospitals, Job satisfaction, Mental health, Motivation, Prevalence, Psychological, Psychology, Health care, Risk factors

## Abstract

Frontline health workers face a significant issue concerning mental health, particularly stress and burnout. Nurses, being among them, grapple with this problem. The study aims to investigate the prevalence and determinants of burnout among nurses. A multicenter cross-sectional study was conducted across twenty two hospitals. A total of nine hundred nurses participated through convenience sampling. Burnout was measured using a single-measurement approach. Both individual and unit-related factors were examined. Over half of the nurses experienced stress, while 7.3% had symptoms of exhaustion. One in every hundred nurses faced a high likelihood of experiencing complete burnout. Job satisfaction, basic salary, motivation, age, incentives, competence, extra responsibilities, and knowledge of quality emerged as significant predictors of burnout. Addressing burnout among nurses requires hospitals to improve job satisfaction, revisit salaries, and foster supportive environments with incentives. Tailored training and ongoing support are crucial for resilience. Prioritizing these strategies is vital for nurses' well-being and sustainable healthcare delivery.

## Introduction

Nurse burnout is a type of psychological, emotional, and physical exhaustion that can occur among nurses due to chronic job stress^1^. Burnout can lead to a range of negative effects, including decreased job satisfaction, reduced quality of patient care, increased rates of medical errors, and increased rates of absenteeism and turnover^2,3^.

The prevalence of nurses’ burnout varies depending on the population and the definition used. However, research suggests that burnout is a significant problem in nursing, with some studies reporting prevalence rates as high as 70%^4^. A meta-analysis analyzed data from 65 studies and found that the overall prevalence of burnout among frontline healthcare workers was 37.4%^5^. A recent study found that the global prevalence of nursing burnout was 30%^6^. Furthermore, burnout was higher among nurses working in certain specialties, such as intensive care units (ICU), emergency departments (ED), and oncology units^6,7^.

Burnout would impact nurses’ turnover intention. A secondary analysis of a 3,9 million registered nurses (RN) survey found that 31.5% of nurses leave their jobs because of burnout, and hospital setting and working more than 20 h per week would increase burnout^8^.

Meanwhile, burnout prevalence among healthcare workers in Indonesia ranges from 22 to 82%^9,10^. Two previous studies showed nurses’ burnout before the pandemic was 34.8%^11^, and during the pandemic, 72.9% of ED nurses felt emotional exhaustion. However, the earlier studies have some limitations, including being conducted in one region or province and having a small sample size.

Some of the factors that contribute to nurses’ burnout include heavy workloads, long shifts, a lack of support from colleagues and supervisors, poor work-life balance, and exposure to traumatic events^2,3^. Nurses in high-stress environments such as the ED, ICU, or oncology units are particularly vulnerable to burnout^6,7^.

Burnout symptoms may include cynicism, emotional exhaustion, depersonalization, and decreased personal accomplishment^12^. Nurses who are experiencing burnout may also have physical symptoms such as headaches, gastrointestinal problems, or sleep disturbances^3^.

The COVID-19 pandemic has significantly strained the healthcare system, and nurses have been on the frontlines of the response. The pandemic has brought new challenges and stressors for nurses, including concerns about personal safety, exposure to the virus, and increased workloads^13,14^.

As a result, many experts predict that the pandemic will have a lasting impact on nurses’ mental health and may lead to higher rates of burnout. A study found that the prevalence of burnout among nurses increased significantly during the COVID-19 pandemic compared to pre-pandemic levels^15^. The study also found that nurses who worked in COVID-19 units and cared for patients who died from COVID-19 had higher rates of burnout.

As the pandemic has stopped in 2022, it is essential to prioritize the mental health and well-being of nurses. This study aimed to explore the prevalence of Indonesian nurses’ burnout and investigate determinants related to it. The study results would reflect the recent situation of nurse burnout in Indonesia, and the hospital could manage the determinants of burnout to increase nurses’ performance.

## Method

### Study design

**Figure 1:**
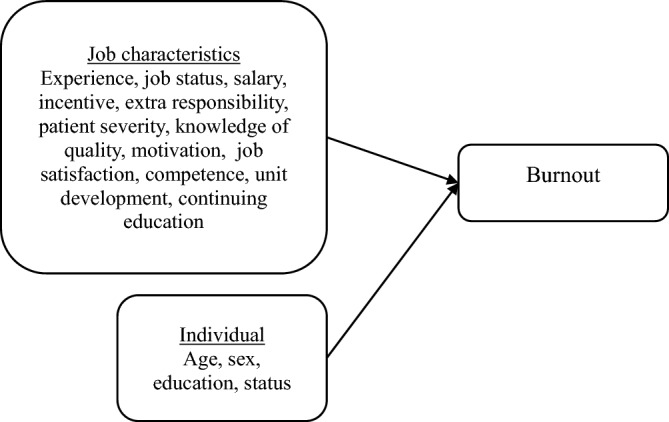
Framework model of burnout.

The study was done through the use of an observational cross-sectional study. The cross-sectional study design is a form of observational study design wherein the investigator concurrently assesses the outcome and the exposures among the study participants^16^. The study used Fig. 1 as the guidance model of burnout.

### Participants and sample size

Nurses from 22 hospitals across Indonesia were involved. The following were the criteria for inclusion: (1) to hold a graduate degree or diploma in nursing from an accredited university and (2) to have worked in an inpatient department (IPD) unit for at least one year. The criteria for excluding participants were professionals who were not currently engaged in paid work when the data were collected.

The sample size was calculated using the Daniel Soper calculator^17^. The anticipated effect size for set B was 0.02, with a statistical power level 0.9. The number of predictors was set at 10 in each set with a probability level of 0.05. The results showed 1,047 samples minimum. The 10% attrition rate was 1,152 samples. The nurses’ response rate was 78.13%.

### Measures

The study was carried out using single-scale burnout. Sociodemographic data (e.g., age, sex, marital status, education) and information concerning the participants’ work (e.g., hospital type, unit, job status, experience, salary, incentive, extra responsibility, patient severity, knowledge of quality, motivation, job satisfaction, competence, unit development, continuing education) were collected using a single scale that constructed by the principal investigator (PI).

Several reports have been written on the subject of the Single Measurement Burnout’s (SMB) psychometric properties. According to the findings of Dolan and colleagues, The SMB successfully detected a favorable outcome for a primary component of emotional exhaustion (EE) in the Maslach Burnout Inventory (MBI), exhibiting a sensitivity of 83.2% and a specificity of 87.4%^18^. Additionally, the newest study found a sensitivity of 53.8% and a specificity of 88.2% for the detection of burnout using the SMB-J. The area under the curve (AUC), which represents the area under the receiver operating characteristic curve, was calculated to be 0.71^19^. Therefore, the diagnostic performance of the SMB indicates that the score has a strong correlation with the score on the MBI EE subscale, and the score may have a high specificity but a low sensitivity for diagnosing burnout based on how it is measured by the MBI. As an alternative to the MBI, the SMB has gained popularity due to its ability to measure emotional exhaustion accurately and its low cost and simple administration.

### Data collection

Questionnaires were sent to one thousand professional nurses across Indonesia. The questionnaires were provided online and in hard copy. The hospital considered it. The online questionnaire was provided through Google form and distributed through the chief nursing officer or headward unit. Hard copies were distributed by research assistants in each hospital. A research assistant sent the copies to the head of the ward and picked them up after one week. It was distributed from July 2022 to February 2023, and nurses contacted and delivered health care directly to the patients involved.

### Data analysis

Statistical analysis was initially performed using Microsoft Excel for Microsoft 365. A *p*-value of 0.05 (two-tailed) was considered to be statistically significant. The measures of descriptive statistics such as mean, standard deviation, frequency, and percentage were used.

Burnout was an ordinal variable. Discrete and continuous variables were used to figure out burnout factors. However, burnout could not pass the parallel test. Based on that, a multinomial linear regression was employed. The assumed significance level was *p* < 0.05. All analyses were performed using the SPSS statistical package version 22 with a significance level of 5%.

### Institutional review board statement

The Declaration of Helsinki principles were adhered to throughout this study. The Indonesian National Research and Innovation Agency (176 /KE.01/SK/8/2022) reviewed and approved the ethical protocol. Additionally, the hospital’s director approved the study. Each participant was supplied with written informed consent prior to enrolment in the study. During data collection, participants could say “no” or “drop out” of the study.

## Result

A total of nine hundred respondents completed the questionnaire (Table 1). Despite there being missing data, we analyzed it all. Univariate and bivariate analyses were done before going to multivariate.

**Table 1:** Descriptive of the results (N = 900).

Variables	Category	Frequency	Percent	Mean (SD)	Min–max
Age	< 26	86	9.6		
	26–30	264	29.3		
	31–35	265	29.4		
	36–40	141	15.7		
	41–45	90	10.0		
	46–50	35	3.9		
	51–55	17	1.9		
	> 55	2	.2		
Sex	Male	223	24.8		
	Female	677	75.2		
	Total	900	100.0		
Education	Diploma	444	49.3		
	Bachelor	45	5.0		
	Bachelor RN	402	44.7		
	Master	7	.8		
	Master specialist	2	.2		
Marital status	Single	181	20.1		
	Married	719	79.9		
	Total	900	100.0		
Hospital type	A/Tertiary	98	10.9		
	B/Secondary	584	64.9		
	C/Primary	218	24.2		
Unit	Non-intensive	689	76.6		
	Intensive	211	23.4		
Job status	PNS/ASN	406	45.1		
	PPPK	25	2.8		
	Permanent	44	4.9		
	Honorer	118	13.1		
	Contract	298	33.1		
	Total	891	99.0		
	Missing	9	1.0		
Experience	As a nurse			10.64 (7.53)	1–35
	Working in the hospital			8.75 (6.90)	1–35
	Working in the unit			5.12 (5.23)	1–33
Basic salary	< Rp2,500,000	442	49.1		
	Rp2,600,000-Rp3,500,000	272	30.2		
	Rp3,600,000-Rp4,500,000	129	14.3		
	Rp4,600,000-Rp5,500,000	46	5.1		
	> Rp5,600,000	1	.1		
	Total	890	98.9		
	Missing	10	1.1		
Incentive	0–500,000	121	13.4		
	500,001–1,000,000	238	26.4		
	1,000,001–1,500,000	158	17.6		
	1,500,001–2,000,000	113	12.6		
	> 2,000,000	126	14.0		
	Total	756	84.0		
	Missing	144	16.0		
Knowledge of quality	Very much	47	5.2		
	A fair amount	485	53.9		
	Some	333	37.0		
	Only little	35	3.9		
	Total	900	100.0		
Extra responsibility	Yes	438	48.7		
	No	462	51.3		
	Total	900	100.0		
Patient severity	Are mostly independent	50	5.6		
	Require some assistance at times	313	34.8		
	Mostly require assistance	403	44.8		
	Bedridden	134	14.9		
	Total	900	100.0		
Motivation	To a very great extent	256	28.4		
	To a fair extent	543	60.3		
	To some extent	79	8.8		
	Only little	14	1.6		
	Not at all	8	.9		
	Total	900	100.0		
Job satisfaction	Very satisfied	423	47.0		
	Enough fair	416	46.2		
	Not really satisfied	53	5.9		
	Not at all	7	.8		
	Total	899	99.9		
	Missing	1	.1		
Competence	Always	615	68.3		
	Sometimes	260	28.9		
	Very seldom	24	2.7		
	Total	899	99.9		
	Missing	1	.1		
Unit development	Very positive	605	67.2		
	Somewhat positive	249	27.7		
	Only little positive	42	4.7		
	Not at all positive	3	.3		
	Total	899	99.9		
	Missing	1	.1		
Continuing education	Yes, very often	303	33.7		
	Yes, at times	506	56.2		
	Never	91	10.1		
	Total	900	100.0		
Burnout	I enjoy my job. I have no symptoms of fatigue	350	38.9		
	I get stressed sometimes, and I don’t always have as much energy as I used to, but I don’t feel exhausted	470	52.2		
	I am completely exhausted and have one or more symptoms of fatigue, such as physical and emotional exhaustion	66	7.3		
	The burnout symptoms that I experienced did not go away. I think a lot about frustration at work	1	.1		
	I feel very tired and often wonder if I can continue	9	1.0		
	Total	896	99.6		
	Missing	4	.4		

The majority of the respondents were 31–35 years old (29.4%), female (75.2%), had a diploma in education (49.3%), and were married (79.9%). A total of 69.4% came from the secondary hospital, and 76.6% worked in the non-intensive rooms. Most nurses had government officer status or were civil servants (PNS/ASN). They have experience as a nurse as of 10.64 (7.53) years and in the unit as of 5.12 (5.23) years. The basic salary of nurses was below two and a half million rupiah (49.1%), and incentives ranged from five hundred rupiah to one million rupiah.

A fair amount of knowledge quality (53.9%), no extra responsibility (51.3%), patient mostly require assistance in the ward (44.8%), a fair extent of motivation (60.3%), feeling very satisfied with their job (47%), feeling always want to update their competence (68.3%), feeling very positive of unit development (67.2%), and want to continue education (56.2%) were the majority. Nurses get stressed sometimes, and they don’t always have as much energy as they used to, but they don’t feel exhausted, which became the dominant finding (52.2%).

Based on the comprehensive multinomial regression of 756 respondents (Table 2), 31.2% variance in burnout could be explained by the predictors, including age, basic salary, incentive, knowing about quality, extra responsibility, motivation, job satisfaction, and competence. The overall percentage of correct classification or accuracy of this model was 62.3%.

**Table 2:** Determinants of burnout.

Effect	Model Fitting Criteria	Likelihood Ratio Tests		
	-2 Log Likelihood of Reduced Model	Chi-Square	df	Sig
Intercept	1176.456	67.340	4	< .001
Basic salary	1122.312	13.195	4	.010
Incentive	1118.889	9.773	4	.044
Knowledge of quality	1121.164	12.048	4	.017
Extra responsibility	1120.899	11.782	4	.019
Motivation	1131.125	22.008	4	< .001
Job satisfaction	1177.036	67.920	4	< .001
Competence	1120.200	11.083	4	.026
Age	1127.176	18.060	4	.001

Table 3 shows that knowledge about quality, extra responsibility, motivation, job satisfaction, and competence influenced nurses to feel stressed. When the nurses became exhausted, the determinants were age, basic salary, motivation, and job satisfaction. Unfortunately, a few respondents filled out burnout level four, “The burnout symptoms that I experienced did not go away; I think a lot about frustration at work,” that could not be measured. Emotional exhaustion was predicted by age and job satisfaction.

**Table 3:** Predictors of burnout in each category.

Burnout		B	Wald	df	Sig	Exp(B)	95% Confidence Interval for Exp(B)	
							Lower Bound	Upper Bound
I get stressed sometimes, and I don’t always have as much energy as I used to, but I don’t feel exhausted	Intercept	− 3.472	41.177	1	.000			
	**Knowledge of quality**	.447	11.581	1	.001	1.564	1.209	2.023
	**Extra responsibility**	− .552	10.162	1	.001	.576	.410	.808
	**Motivation**	.354	6.998	1	.008	1.425	1.096	1.852
	**Job satisfaction**	.913	35.715	1	.000	2.492	1.847	3.362
	**Competence**	.573	10.289	1	.001	1.774	1.250	2.519
I am completely exhausted and have one or more symptoms of fatigue, such as physical and emotional exhaustion	Intercept	− 6.630	37.543	1	.000			
	**Basic salary**	.540	10.072	1	.002	1.716	1.230	2.396
	**Motivation**	.974	19.502	1	.000	2.648	1.719	4.079
	**Job satisfaction**	1.762	47.135	1	.000	5.827	3.523	9.637
	**Age**	− .386	6.992	1	.008	.680	.511	.905
I feel very tired and often wonder if I can continue	Intercept	4.453	.068	1	.795			
	**Job satisfaction**	1.816	9.738	1	.002	6.147	1.965	19.231
	**Age**	− 1.521	5.992	1	.014	.218	.065	.738

*Reference group: I enjoy my job. I have no symptoms of fatigue.

*Insignificant categories and factors are deleted.

**Figure 2:**
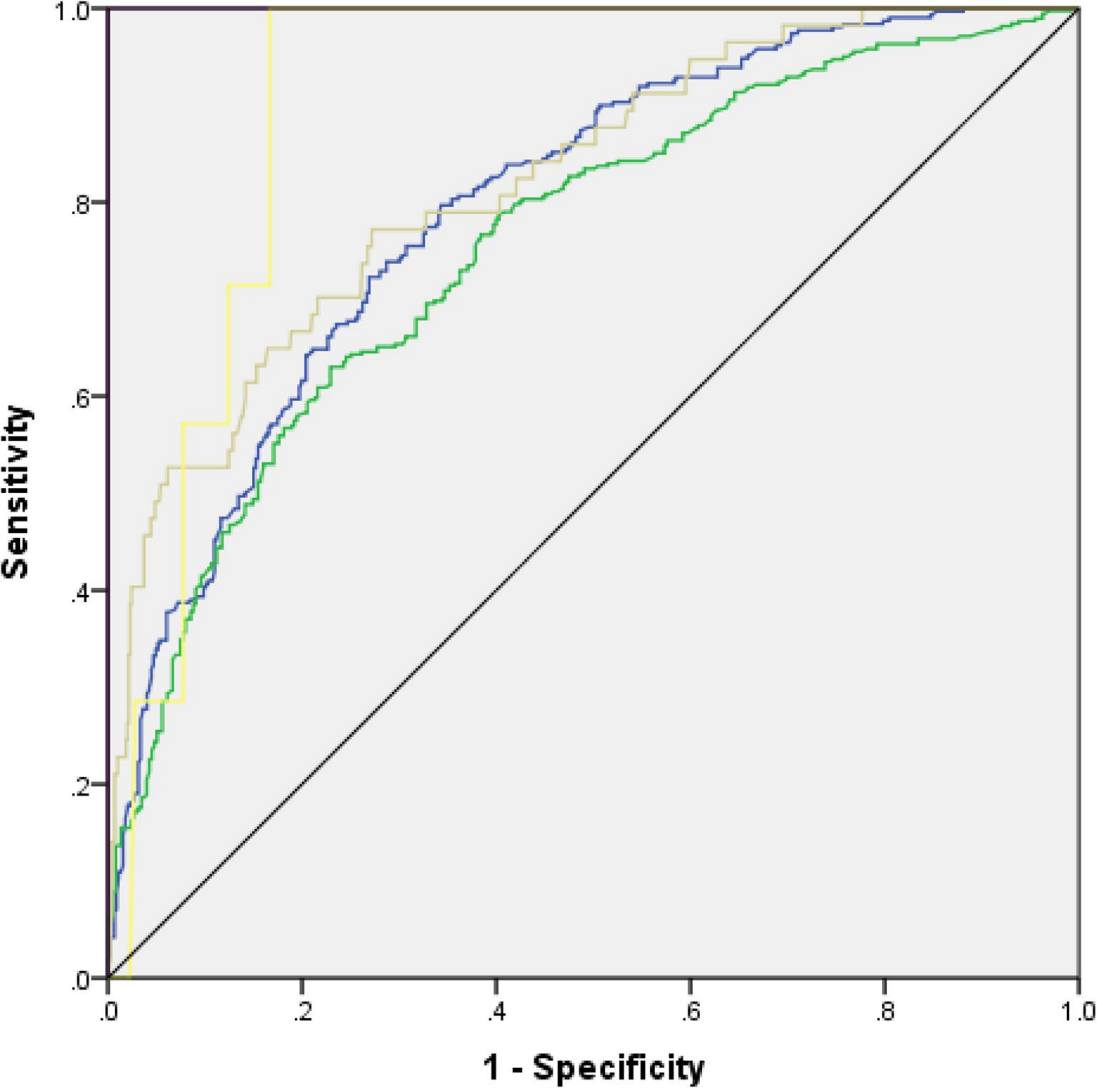
Receiver operating characteristics curve (ROC) of burnout model.

Significant results were shown for each category by the amount of area under the curve (Fig. 2). The stage with the best results was four, which received 1.00, followed by stage five, which received 0.905, and stage three, which received 0.821. The results for the AUC obtained in stage one and stage two were, respectively, 0.796 and 0.756. As a result, the classifier that was used in this research is more likely proper and correct.

Based on the results of the importance normalized variables, job satisfaction was the highest importance variable (100%), followed by motivation (79.3%). In the next place were age (78.5%), incentive (63.7%), basic salary (46.1%), and extra responsibility (39.6%). Knowledge of quality and competence became the last factors, 36.4%, and 35.6%, respectively.

## Discussion

The prevalence of stressed Indonesian nurses was more than fifty percent, and 7.3% of nurses felt one of the exhaustion symptoms. One in one hundred nurses in Indonesia has a chance to feel totally burned out. The responsible predictors of this situation were job satisfaction, basic salary, motivation, age, incentive, competence, extra responsibility, and knowledge of quality.

The finding is supported by the previous studies showed that the prevalence of nurses’ stress in Iran, Australia, and Globally was 69%, 41.2%, and 42%, respectively^20–22^. Additionally, a former study supports our burnout finding that revealed global nurses burnout was 11.23%^23^. On the contrary, another studies found higher that the global and United States prevalence of nurses' burnout was approximately 30%^6,8^. Nurse leader could use these findings to manage their team. Stress and burnout could impact their productivity^24^.

Job satisfaction has a significant impact on burnout. All stages of burnout were affected. The result is supported by a meta-analysis study that found job satisfaction to have the most significant effect^25^. Another previous study aligns with this finding that nurses’ job satisfaction significantly impacts burnout^26^. Hospitals could consider an intervention to maintain nurse job satisfaction. Low job satisfaction and burnout would affect the nurse’s performance and productivity^27^. Empowerment strategy could be used by hospitals to increase nurses’ satisfaction^28^.

Motivation significantly impacted Indonesian nurses’ burnout. The previous study supported this finding, indicating that motivation significantly influences emotional exhaustion, depersonalization, and personal accomplishment^29^. Furthermore, nurses’ intrinsic motivation in the nursing home significantly predicted burnout^30^. Personal or intrinsic motivation is essential to support nurses’ conditions. When nurses lose motivation, burnout increases and decreases their performance and productivity.

The younger nurses have more significant burnout than the older nurses. The meta-analysis study^31^ found older nurses showed lower emotional exhaustion and depersonalization than younger nurses. It found that sex and marital status moderated age and burnout. On the contrary, a study from the United States showed that older age and lower physical and psychological ability would increase burnout^32^. Another study revealed that women aged 40–50, working full time, being married, and having children are the most vulnerable to burnout^33^. The different results might be due to different sexes, marital statuses, numbers of children, job statuses, workloads, experiences, basic salaries, and incentives.

Incentive is a part of extrinsic motivation for nurses. A higher incentive would stimulate lower burnout and better performance. The study from Greece found that the incentive system is essential to increase the number of nurses initiating or participating in the innovative program^34^. A qualitative study revealed that insufficient welfare and financial facilities lead nurses to burnout^35^. In addition, a study from China showed that organizational support increases extrinsic motivation. There is a limited study that explores nurses’ incentives related to burnout. Further study would be needed.

Basic salary has become the fifth important predictor influencing burnout. Two previous studies found that salary significantly affects burnout^36,37^. The basic monthly minimum salary in Indonesia is IDR 1,812,935.43 (USD 123.15) – IDR 4,461,854 (USD 303.09), with an average of IDR 2,726,828.34 (USD 185.23) (USD 1 = IDR 14,721)^38^. The situation in each province is different. It may be that some nurses feel the salary cannot cover the family’s needs or is incomparable with the workload.

Extra responsibility would give nurses an extra workload and task lists. Two qualitative studies found extra responsibility related to nurse burnout^39,40^. Extra responsibility was related to the nursing shortage, and nurses have no extra payment for doing it^41^. Additionally, a study from China found nurses suffered from an extra workload and intense burnout during hospital-grade re-evaluation. Hospitals could provide cheap and useful self-therapy to the nurses that would keep control of their mental health^42^.

Competence and knowledge of quality care are related to how nurses consider their practice to provide good quality care to patients. Nurses would like to meet the standard or better to maintain the quality^43^. However, many problems in real-life situations could impact nurses’ feelings. A systematic review showed that nurses and doctors, healthcare providers and patients, and male and female health workers have different perspectives on what causes stress and what constitutes high-quality care, which is recognized and contributes to the complexity of the healthcare industry^44^. That indicated health professionals and patients react to stressful and potentially dangerous situations in a variety of ways, such as by questioning the efficacy and fairness of health policy and institutional management, blaming others and placing blame outside of their control, assuming a victim role, and challenging hospital hierarchies and injustice, among others.

## Implication

The study has a broad impact on hospital management. Hospital upper-class management, supervisors, and ward managers/first-line managers could consider the predictors that impact nurses’ feelings. Feelings of burnout could affect their performance and lead to low-quality nursing care. Keeping nurses satisfied and motivating them continuously will positively impact their minds and well-being. Additionally, older nurses could be role models in mind and well-being. Additionally, older nurses could be role models to young or newly graduated nurses to help them face working problems.

The burnout situation could be understood by newly graduated nurses. They can prepare and tackle any situation. However, a complex system of organization might be different in each region or country. The preparation might be extra according to the context, and our study findings could be considered.

## Limitation

The measurement of only one item at a time could be restrictive. A large number of researchers were unable to reach an agreement on the single-item measurement. There was a significant number of missing data in certain variables due to the number of questions asked during the research. The nurse’s situation might have varied from hospital to hospital and region to region. The obstacles in the workplace were beyond our ability to overcome. In addition, a larger sample size (one greater than one thousand) and an appropriate number of questions might be the best solution to cut down on missing data and cells. It would be beneficial to lengthen the amount of time spent on research for multicentre national studies.

## Conclusion

A majority of nurses experience feelings of stress, with over 50% reporting such sentiments. Additionally, a notable proportion of nurses, specifically 7.3%, reported experiencing symptoms indicative of exhaustion. Approximately 1% of nurses in Indonesia are at risk of experiencing complete burnout.

The degree to which Indonesian nurses are satisfied with their jobs, their basic salaries, their levels of motivation, their ages, the incentives they receive, their levels of competence, the additional responsibilities they take on, and their knowledge of quality are all essential predictors in their stress levels. The degree of job satisfaction is the most crucial factor in determining whether or not an employee will burn out, followed by their base pay. In order to keep nurses happy in their jobs, hospitals might want to consider implementing an intervention. Additionally, the importance of one’s own personal drive cannot be overstated. A higher incentive system can stimulate a lower burnout rate and better performance among nurses, as the incentive is part of the extrinsic motivation nurses receive from their employers. Extra responsibilities can easily lead to burnout, and a nurse’s level of competency and knowledge of quality care are directly related to how she approaches her practice in order to provide patients with good-quality care.

These predictors could be examined in greater depth in future research, specifically in relation to Indonesian nurses. Hospitals should prioritize the preparation of a more effective system to boost the job satisfaction and motivation of nurses. It is of the utmost importance to maintain their mental health and place nurses in accordance with their ages. In addition, the base salary, the incentive, and the additional responsibility could be evenly distributed. A lack of balance would increase the risk of burnout. In conclusion, but certainly not least, it is essential that nurses’ skills and knowledge be kept current and that they be given the opportunity to put those updates into practice.

## Data Availability

The datasets generated and analyzed during the current study are not publicly available due to hospital requirements but are available from the corresponding author upon reasonable request.
